# Le tératome cervical: à propos de 2 cas

**Published:** 2012-07-30

**Authors:** Mohamed Rami, Abdelhalim Mahmoudi, Aziz ElMadi, Khalid, Khattala, Abderrahmane Afifi, Youssef Bouabdallah

**Affiliations:** 1 Service de chirurgie pédiatrique, CHU Hassan II, Fès, Maroc

**Keywords:** Tératome cervical géant, nouveau né, chirurgie, Cervical teratoma, newborn, surgery

## Abstract

Le tératome est une tumeur embryonnaire généralement localisé au niveau sacro-coccygien. La localisation cervicale est rare, et ne représente que 3%, elle est associée à un fort taux de mortalité arrivant jusqu’à 80 % à la période néonatale du fait de l'obstruction des voies aériennes. L'immaturité n'est pas un signe de malignité quand la tumeur est prise en charge à la période néonatale. Nous rapportons 2 cas colligés au service de chirurgie pédiatrique du CHU HASSAN II de Fès, en mettant en relief qu'il existe encore un manque de diagnostic anténatal, malgré l'amélioration de la prise en charge des nouveaux nés.

## Introduction

Du mot grec tératos = monstre, les tératomes sont des tumeurs malformatives dérivant de la transformation des cellules germinales. La localisation cervicale est rare, et nécessite une prise en charge multidisciplinaire. Le diagnostic anténatal est primordial vu le risque de détresse respiratoire. Il est encore peu réalisé dans notre contexte, et retarde de ce fait la prise en charge.

## Observation

### Observation 1

Il s'agit d'un nouveau-né de sexe masculin, sans antécédents particuliers, admis à J3 de vie pour une masse cervicale droite. L'examen trouve un nouveau-né tonique, réactif, pouls = 92 battement/min, une fréquence respiratoire à 32 Cycles/min, sans cyanose, ni dyspnée, une masse rénitente de 10 cm environ, indolore avec télangiectasies. Le bilan biologique est revenu normal, et les taux de ßHCG et aFP négatifs. Le scanner cervical a objectivé une masse hypodense contenant des travées et des calcifications, sans envahissement des vaisseaux ni de la trachée. Le traitement a consisté en l'exérèse chirurgicale totale de la masse, qui n'envahissait pas les structures adjacentes. L’étude anatomopathologique était en faveur d'un tératome immature multi-tissulaire, avec 10 % de blastème, et un tissu nerveux immature. L’évolution est bonne, le patient a été perdu de vue à 4 mois du postopératoire.

### Observation 2

Il s'agit d'un nouveau-né de 23 jours, sans antécédents particuliers, qui présente depuis la naissance une masse cervicale antérolatérale droite, augmentant progressivement de volume. La famille rapporte également la notion de dyspnée et de cyanose notamment lors des tétées. L'examen trouve un nourrisson en bon état général, avec une masse cervicale d'environ 8 cm étendue depuis le creux sus-claviculaire jusque sous le menton, sans signes inflammatoires, ni circulation veineuse collatérale. La masse est fixe par rapport au plan profond, indolore, polylobée, et s’étend latéralement arrivant au muscle sterno-cléÏdo-mastoïdien. Le bilan biologique montrait une élévation de l'alpha-fœtoprotéine à 71,35 ng/mL. Le scanner cervical a montré une masse de 95/60 mm, tissulaire contenant des calcifications se rehaussant modérément, refoulant sans envahir les organes et vaisseaux voisins. Le traitement a consisté en l'exérèse totale de la masse. L'examen histologique est revenu en faveur d'un tératome mature. Les suites étaient simples, avec négativation du taux de l'alpha- fœtoprotéine. Le recul est de 2 ans.

**Figure 1:**
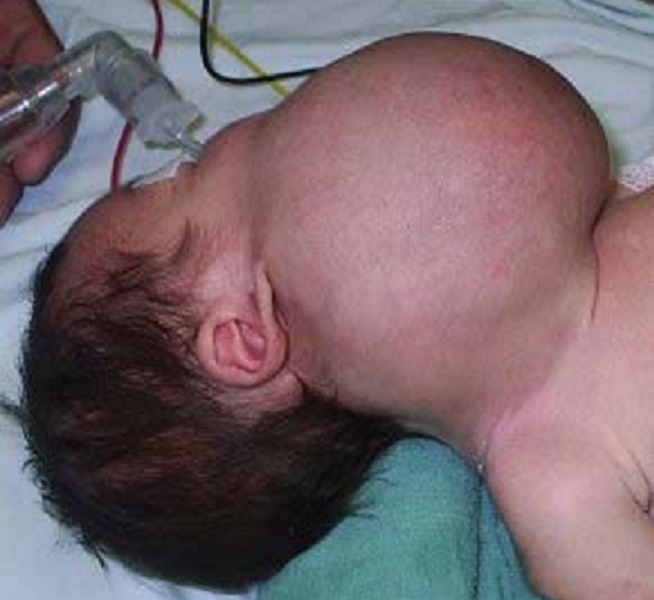
Masse laterocervicale chez le patient 1

**Figure 2:**
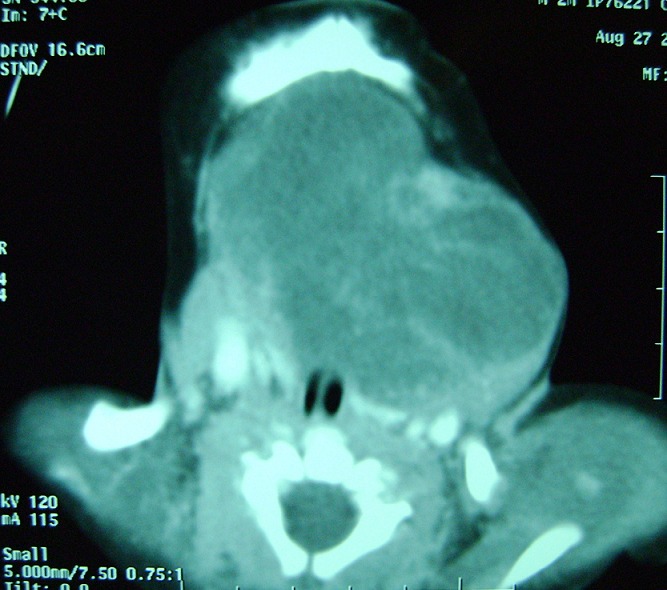
Scanner cervicofacial montrant le tératome chez le patient 2

**Figure 3:**
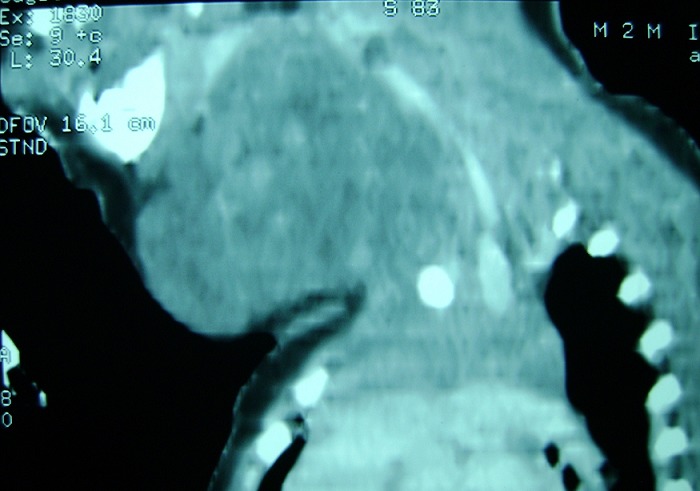
Scanner cervicofacial en coupe sagittale chez le même patient

**Figure 4:**
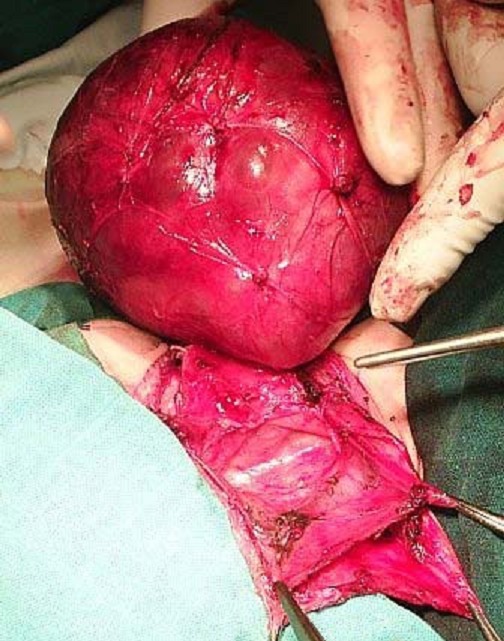
Image peropératoire du patient 1

Les Figure 1, Figure 2, Figure 3,et Figure 4 sont associées aux deux observations.

**Figure 5:**
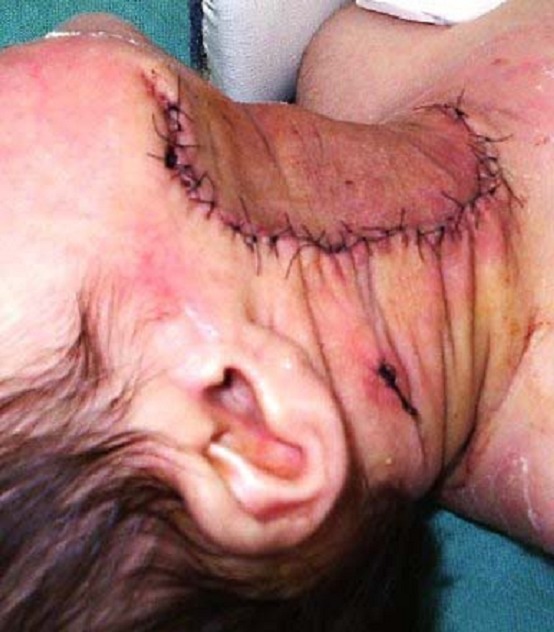
Aspect postopératoire chez le patient 1

## Discussion

Du grec tératos (monstre), les tératomes sont des tumeurs malformatives dérivant de la transformation des cellules germinales multipotentes. Ils sont composés de tissus ectodermiques, endodermiques et mésodermiques en proportions variables d'o[ugrave] le terme de tumeur embryonnaire [1, 2].

Il s'agit d'une tumeur rare, 1/40000 naissances. La localisation cervicale représente 1,5 à 5 % de toutes les localisations [3]. Il existe une nette prédominance féminine (3/4 des cas). Son volume, empêchant la croissance normale du fœtus, peut être responsable d'une hypotrophie ou de prématurité. Les cellules germinales ou gonocytes primaires migrent au dépend du sac vitellin au cours des 1ères semaines de la vie intra-utérine, et vont coloniser le cordon sexuel formant des gonades primitives indifférenciées. Elles peuvent s'arrêter le long de leur migration pour se transformer et former une tumeur germinale bénigne ou maligne, celles-ci peuvent ainsi se localiser de la tête au coccyx de l'enfant [3, 4].

Le tératome est une tumeur très hétérogène, kystique avec des parties solides. On peut retrouver des cheveux, des fragments osseux ou cartilagineux, des structures parfois organoïdes. Il est nécessaire de couper la totalité de la tumeur et de faire des prélèvements multiples pour ne pas manquer une zone indifférenciée, maligne dont la présence peut changer le pronostic [3, 5].

Le diagnostic anténatal à l’échographie est possible dès le 2ème trimestre devant un hydramnios, mais surtout si l'on visualise une masse contenant des calcifications. On peut alors compléter par une IRM fœtale, celle-ci renseignera sur le degré de compression des voies aériennes supérieures. Le diagnostic anténatal permet de préparer la prise en charge du nouveau-né par une équipe multidisciplinaire, devant le risque de détresse respiratoire, mais également de prévenir une dystocie à l'accouchement, ou une rupture de la tumeur. Malheureusement, dans notre contexte il est encore peu réalisé, et le diagnostic n'est fait qu’à la naissance [3, 5]. Ainsi une détresse respiratoire, causée par la tumeur serait fatale pour le nouveau-né. Les deux cas de notre étude n'avaient heureusement peu ou pas de signes respiratoires, facilitant leur prise en charge. A la naissance le scanner ou mieux, l'IRM, permettent une bonne étude de la tumeur, la présence de calcifications, ses caractéristiques et de ses rapports avec les organes et vaisseaux. Le bilan sera compléter par le dosage de l'a fœtoprotéine, qui sera refait après exérèse [5, 6].

Le pronostic est surtout respiratoire, lorsque le volume de la masse est important et comprime les voies aériennes. Ainsi le diagnostic anténatal permet une bonne prise en charge. Deux techniques de ventilation artificielle dès la naissance avant de clamper le cordon ombilical sont décrites, par un césarienne programmée : EXIT (ex- utero intra partum technique) où la tête du fœtus est extériorisé pour permettre l'exploration des voies aériennes, et réaliser une intubation voire une trachéotomie si nécessaire; OOPS (opération on placenta support) où le nouveau-né est extériorisé en totalité et mis une table chirurgicale pour l'examen des voies aériennes. Ces techniques nécessitent une relaxation utérine maximale, pouvant être responsable d'hémorragie utérine grave, et de complications pour le nouveau-né à type : de thrombopénie, d'ascite ou de pleurésie [2, 6, 7]. La chirurgie sera réalisé après une bonne mise en condition, sauf s'il y a dégradation de l’état général. L'exérèse totale de la tumeur est souvent aisée, vu qu'elle possède un plan de clivage par rapport aux tissus et organes de voisinage [6, 7]. Ce qui a été le cas pour nos deux patients. Le pronostic dépens de la gravité des signes respiratoires, et des signes de malignité. Il est généralement bénin chez le nouveau-né.

## Conclusion

Le tératome cervical, est une localisation rare. Le traitement est chirurgical. Le diagnostic anténatal permet de mieux prendre en charge ces patients du fait du risque de détresse respiratoire à la naissance par compression. Le pronostic dépens essentiellement des signes respiratoires, et de la malignité ou non.
